# Epoxide hydrolase 3 (*Ephx3*) gene disruption reduces ceramide linoleate epoxide hydrolysis and impairs skin barrier function

**DOI:** 10.1074/jbc.RA120.016570

**Published:** 2021-01-21

**Authors:** Matthew L. Edin, Haruto Yamanashi, William E. Boeglin, Joan P. Graves, Laura M. DeGraff, Fred B. Lih, Darryl C. Zeldin, Alan R. Brash

**Affiliations:** 1 Division of Intramural Research, NIEHS/NIH, Research Triangle Park, North Carolina, USA; 2 Department of Pharmacology and the Vanderbilt Institute of Chemical Biology, Vanderbilt University School of Medicine, Nashville, Tennessee, USA; 3 Department of Dermatology and Allergology, Juntendo University Graduate School of Medicine, Bunkyo-ku, Tokyo, Japan

**Keywords:** 12*R*-lipoxygenase, ichthyosis, epidermis, transepidermal water loss, EPHX1, EPHX2, EPHX3, LC-MS, epoxyalcohol, skin barrier function, AA, arachidonic acid, DiHOMEs, dihydroxyoctadecamonoenoic acids, DMP, dimethoxypropyl, EETs, epoxyeicosatrienoates, EpFAs, epoxy fatty acids, EPHX, epoxide hydrolase, EpOMEs, epoxyoctadecamonoenoic acids, PFB, pentafluorobenzyl, LA, linoleic acid, sEH, soluble epoxide hydrolase, TEWL, transepidermal water loss

## Abstract

The mammalian epoxide hydrolase (EPHX)3 is known from *in vitro* experiments to efficiently hydrolyze the linoleate epoxides 9,10-epoxyoctadecamonoenoic acid (EpOME) and epoxyalcohol 9*R*,10*R*-*trans*-epoxy-11*E*-13*R*-hydroxy-octadecenoate to corresponding diols and triols, respectively. Herein we examined the physiological relevance of EPHX3 to hydrolysis of both substrates *in vivo*. *Ephx3^−^^/^^−^* mice show no deficiency in EpOME-derived plasma diols, discounting a role for EPHX3 in their formation, whereas epoxyalcohol-derived triols esterified in acylceramides of the epidermal 12*R*-lipoxygenase pathway are reduced. Although the *Ephx3^−^^/^^−^* pups appear normal, measurements of transepidermal water loss detected a modest and statistically significant increase compared with the wild-type or heterozygote mice, reflecting a skin barrier impairment that was not evident in the knockouts of mouse microsomal (EPHX1/microsomal epoxide hydrolase) or soluble (EPHX2/sEH). This barrier phenotype in the *Ephx3^−^^/^^−^* pups was associated with a significant decrease in the covalently bound ceramides in the epidermis (40% reduction, *p* < 0.05), indicating a corresponding structural impairment in the integrity of the water barrier. Quantitative LC-MS analysis of the esterified linoleate-derived triols in the murine epidermis revealed a marked and isomer-specific reduction (∼85%) in the *Ephx3^−^^/^^−^* epidermis of the major trihydroxy isomer 9*R*,10*S*,13*R*-trihydroxy-11*E*-octadecenoate. We conclude that EPHX3 (and not EPHX1 or EPHX2) catalyzes hydrolysis of the 12*R*-LOX/eLOX3-derived epoxyalcohol esterified in acylceramide and may function to control flux through the alternative and crucial route of metabolism *via* the dehydrogenation pathway of SDR9C7. Importantly, our findings also identify a functional role for EPHX3 in transformation of a naturally esterified epoxide substrate, pointing to its potential contribution in other tissues.

The mammalian epoxide hydrolases epoxide hydrolase (EPHX)1 (microsomal EH, microsomal epoxide hydrolase) and EPHX2 (soluble EH; soluble epoxide hydrolase [sEH]) are especially well characterized for their participation in xenobiotic bioactivation and inactivation (1, 2). The mouse gene knockouts were reported in 1999 and 2000, and this helped confirm their leading role in transformation of xenobiotic epoxides (3, 4). Both microsomal epoxide hydrolase and sEH also contribute to hydrolysis of cytochrome P450-derived fatty acid epoxides, such as the arachidonic acid (AA)-derived epoxyeicosatrienoates (EETs), to less biologically active dihydroxy derivatives (DHETs) (5). sEH-deficient mice show protection in animal models of inflammation, hypertension, and ischemia, which resulted in the development of sEH inhibitors for treatment of human cardiovascular diseases and pain (6, 7).

More recently, gene sequencing revealed the existence of two additional mammalian epoxide hydrolases. Currently, little is known of the product of the EPHX4 gene, other than its almost exclusive expression in the brain (2, 8). EPHX3 is better characterized. Decker *et al.* (9) and the Human Protein Atlas (www.proteinatlas.org) show that EPHX3 is highly expressed in proximal digestive tract, bone marrow, lymphoid tissues, and the skin. While EPHX3 is thought to be a membrane-bound hydrolase, a truncated soluble form was shown to metabolize linoleic acid (LA)-derived epoxides (epoxyoctadecamonoenoic acids; EpOMEs) to dihydroxyoctadecamonoenoic acids (DiHOMEs) at a high rate in *in vitro* assays (9). While less well studied than EETs and DHETs, EpOMEs and DiHOMEs are emerging as important proinflammatory, cytotoxic, and tumor-promoting oxylipins (10, 11). EpOMEs and DiHOMEs are abundant in the skin where altered metabolism may contribute to conditions such as ichthyosis or psoriasis (12, 13); however, the murine knockout of EPHX3 did not alter the levels or metabolism of LA- or AA-derived epoxy fatty acids (EpFAs) *in vivo* and displayed no overt phenotype (14).

In 2008, Ala *et al.* (15) reported on an extensive mRNA expression analysis that implicated EPHX3 as a potential ichthyosis-related gene. Their method relied on the principle that genes implicated in similar phenotypes tend to share similar expression profiles and that mRNA expression analysis on massive microarray data sets in mouse and human could identify disease-specific gene candidates. The implication that EPHX3 expression could be associated with ichthyosis suggested it is involved in skin barrier function. The ichthyosis phenotype, a congenital scaly skin, entails a hyperproliferation of the epidermis, usually associated with a defect in the water permeability barrier of the epidermis (16, 17). Multiple genes involved in lipid metabolism are implicated in skin barrier function (18). In most cases they were identified through a rare familial deficiency tracked by geneticists, and usually the mouse phenotype entails neonatal lethality associated with uncontrollable transepidermal water loss (TEWL) (17, 19, 20). Such is the case with inactivation of genes of the 12*R*-lipoxygenase pathway of acylceramide transformation in the epidermis, including ALOX12B (12*R*-LOX), ALOXE3 (eLOX3), and the short-chain dehydrogenase-reductase SDR9C7 ((20, 21, 22), and see below).

The 12*R*-lipoxygenase (12*R*-LOX) pathway of skin barrier formation includes the synthesis of a lipid epoxide and its hydrolysis by a previously unidentified epoxide hydrolase (23). The pathway is initiated by 12*R*-LOX catalyzed oxygenation of the essential fatty acid linoleate (C18:2ω6) esterified in the skin-specific acylceramide Cer-EOS (Esterified Omega-hydroxy-Sphingosine), forming the linoleate 9*R*-hydroperoxide as the ceramide ester, Figure 1. A second ichthyosis-related gene product, epidermal lipoxygenase-3 (eLOX3), acting as an hydroperoxide isomerase, then converts the 9*R*-hydroperoxide to a 9*R*,10*R*-*trans*-epoxy-13*R*-hydroxy epoxyalcohol. One of two potential pathways further convert the epoxyalcohol. The short-chain dehydrogenase-reductase SDR9C7 mediates formation of an epoxy-ketone, which appears essential for protein binding and formation of the corneocyte lipid envelope (22). Alternatively, the epoxyalcohol can undergo hydrolysis and reversal of configuration at C-10 *via* an uncharacterized epoxide hydrolase, to the Cer-EOS linoleate ester 9*R*,10*S*,13*R*-trihydroxy-octadecenoate, Figure 1. Mouse gene knockout of SDR9C7 is neonatal lethal, as typical of ichthyosis-related genes, whereas, as noted, EPHX3 gene deficiency showed no overt phenotype (14). Biochemical analysis of potential epoxide hydrolases converting the linoleate-derived 9*R*,10*R*-*trans*-epoxy-13*R*-hydroxy epoxyalcohol to the 9*R*,10*S*,13*R*-triol identified EPHX3 as a candidate for catalyzing the *in vivo* hydrolysis. sEH hydrolyzed 14,15-EET at twice the rate of the linoleate epoxyalcohol, whereas human or murine EPHX3 hydrolyzed the 9*R*,10*R*,13*R*-epoxyalcohol at 31-fold and 39-fold higher rates, respectively (24).

**Figure 1:**
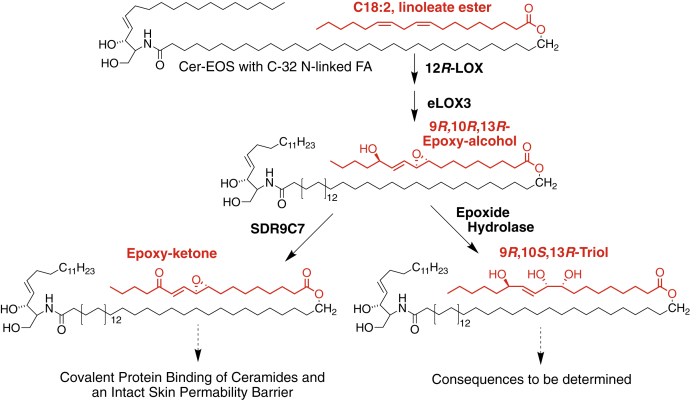
**The 12*R*-lipoxygenase pathways in epidermal barrier formation**.

Herein, we investigate the role of EPHX3 in the regulation of linoleic acid epoxide hydrolysis and skin barrier function. We examine the relative contributions of EPHX1, EPHX2, and EPHX3 in EpOME hydrolysis through the generation of *Ephx1/2/3*-deficient mice. In addition, to uncover the role of epoxyalcohol hydrolysis in the 12*R*-LOX epidermal pathway, we examined the effects of EPHX3 deficiency on the *in vivo* linoleate ester transformations and subsequent effects on the skin barrier permeability.

## Results

### Mouse epoxide hydrolases involved in hydrolysis of soluble linoleate epoxides

**Figure 2:**
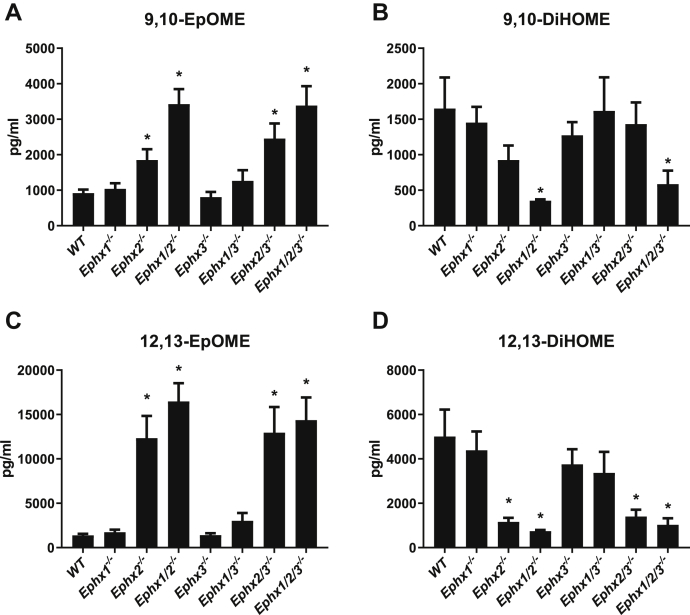
**Plasma concentrations of linoleate-derived epoxides (EpOMEs) and diols (DiHOMEs) from epoxide hydrolase-deficient mice.** Mouse plasma levels of 9,10-EpOME (*A*) 9,10-DiHOME (*B*) 12,13-EpOME (*C*) and 12,13-DiHOME (*D*) in relation to epoxide hydrolase genotype. ±SEM, n = 5 to 6/genotype, ∗*p* < 0.05 *versus* WT.

A truncated/solubilized version of EPHX3 previously demonstrated rapid hydrolysis of linoleate-derived 9,10-epoxyoctadecamonoenoic acid (9,10-EpOME) *in vitro* (9); however, *Ephx3*^*−/−*^ mice show no change in hydrolysis of EpOMEs to DiHOMEs. Recently, the substantial *in vivo* role of EPHX1 in EpFA hydrolysis was unmasked only after generation of *Ephx1/2*^*−/−*^ mice (5). Disruption of both *Ephx1* and *Ephx2* abolishes hydrolysis of most AA, EPA, and DHA-derived epoxides *in vivo*; however, plasma from *Ephx1/2*^*−/−*^ mice reveal substantial residual DiHOME formation (5). To determine whether EPHX3 is responsible for the remaining EpOME hydrolysis, we examined EpOME and DiHOME levels in the plasma from *Ephx1/2/3*^*−/−*^ mice. Disruption of *Ephx2* or *Ephx1/2* strongly reduces the concentrations of the 9,10- and 12,13-DiHOME; however, additional disruption of *Ephx3* fails to further reduce formation of either DiHOME (Fig. 2). Reciprocal increases in the plasma levels of the substrates 9,10- and 12,13-EpOME are not further increased by additional *Ephx3* disruption. Moreover, analysis of AA, DHA, and EPA-derived oxylipins reveals no role for EPHX3 in EpFA hydrolysis (Table S1).

### Epoxide hydrolase gene deficiency and skin water permeability in neonatal pups

**Figure 3:**
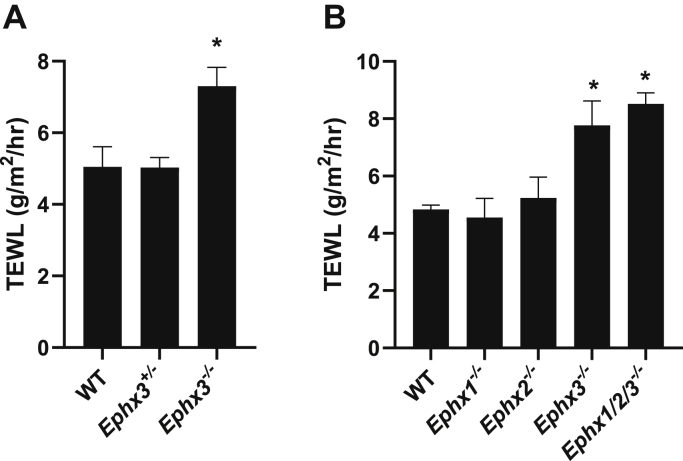
**Effect of *Ephx3* deficiency on transepidermal water loss (TEWL) and in combination with deficiencies in *Ephx1* and *Ephx2*.***A*, TEWL of dorsal skin was determined on postnatal day 2 from wild-type (WT), heterozygous (*Ephx3*^*+/*^^*−*^), and homozygous null (*Ephx3*^*−*^^*/*^^*−*^) *Ephx3*-deficient pups (±SEM, n = 7–19 each genotype, ∗*p* < 0.05 *versus* WT). *B*, effects of individual knockouts and in combination. *C*, comparison of TEWL in wild-type, *Ephx3^−^^/^^−^*, and the triple knockout *Ephx1/2/3*^*−*^^*/*^^*−*^. ±SEM, n = 5 to 37/genotype ∗*p* < 0.05 *versus* WT.

To assess the effects of epoxide hydrolase deficiency on a parameter of skin barrier function, TEWL measurements were recorded on neonatal pups with single or triple knockouts of *Ephx1*, *Ephx2*, and *Ephx3*. The results demonstrate a significant increase in water loss only with gene combinations that include the *Ephx3*^*−/−*^ genotype (Fig. 3*A*). This is not mimicked by *Ephx1*^*−/−*^ or *Ephx2*^*−/−*^ individually, and the combined triple knockout does not further enhance the effects of the *Ephx3*^*−/−*^ genotype (Fig. 3*B*). Parsing out the results with *Ephx3*^*−/−*^ genotypes only, the results indicate no effect in heterozygous mice and reveal a modest increase of about 45% in water permeability in the knockout pup epidermis (Fig. 3*A*). This is in contrast to the drastic increases in TEWL observed in ichthyosis gene knockouts, which are usually measured in the order of tenfold higher than wild-type and are associated with neonatal fatality (*e.g.*, (22, 25, 26, 27)), whereas all the *Ephx3^−^^/^^−^* pups survive with no apparent problems of desiccation.

### Analysis of covalently bound ceramides

**Figure 4:**
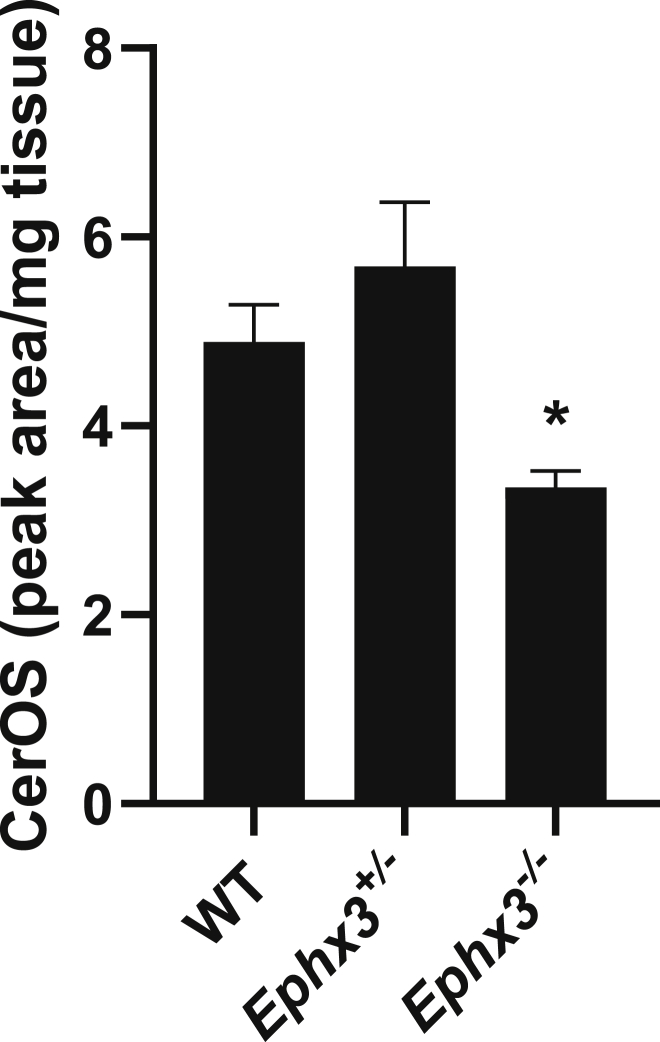
**Quantitation of covalently bound ceramides in epidermis of *Ephx3* genotypes.** Relative abundances of covalently bound ceramides in epidermis of wild-type (WT), heterozygous (*Ephx3^+/^^−^*), and homozygous null (*Ephx3^−^^/^^−^*) Ephx3-deficient mice. ± SEM, n = 3/genotype, ∗*p* < 0.05 *versus* WT.

The ceramides covalently bound in the outer epidermis form a lipid coating on the proteinaceous corneocyte envelope that is vital to the structural integrity of the water permeability barrier. This covalent lipid coating, known as the corneocyte lipid envelope, is almost eliminated in several of the gene inactivations associated with ichthyosis in humans and the lethality caused by uncontrollable water loss in neonatal mice. Of direct relevance to the 12*R*-LOX pathway, >90% loss of covalent binding occurs with mouse knockouts of 12*R*-LOX, eLOX3 or Sdr9c7 (22, 28, 29). Covalent binding is measured after extensive washing of the epidermal extracts with chloroform/methanol to remove noncovalently bound lipids, followed by mild alkali treatment to release esterified lipids and LC-MS analysis of the omega-hydroxy ceramide (Cer-OS) levels. The results of Cer-OS analysis on the mouse *Ephx3*^*−/−*^ epidermal samples induced a small but statistically significant reduction of covalently bound Cer-OS in the *Ephx3*^*−/−*^ pup epidermis (Fig. 4). This corresponds with the modest increase in TEWL and compromised barrier function.

### Analysis of esterified linoleate triols in Ephx3-deficient mouse epidermis

**Figure 5:**
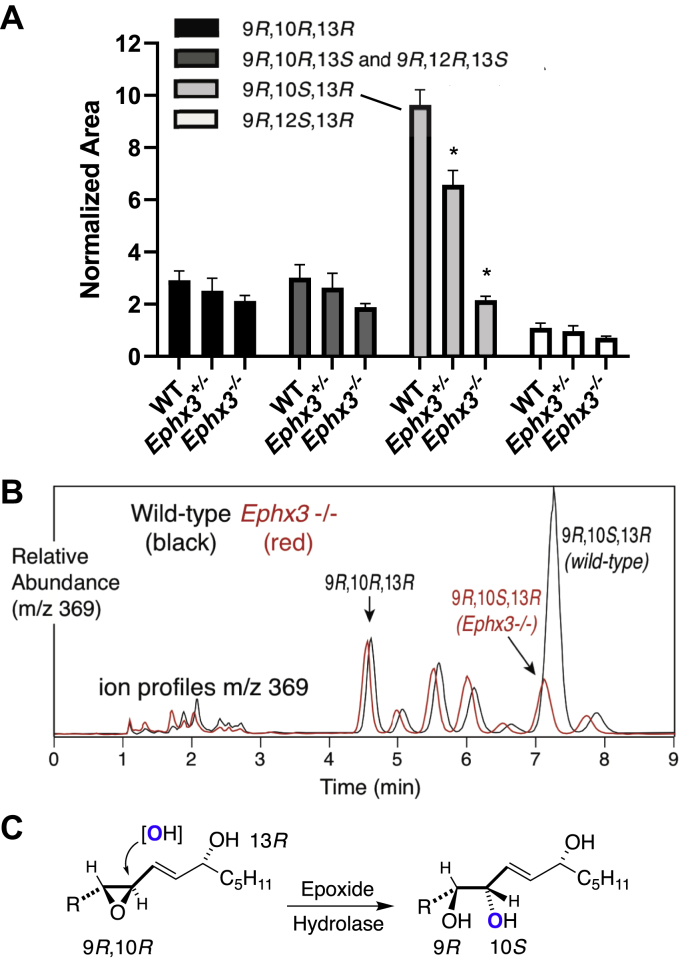
**Effect of *Ephx3* deficiency on esterified linoleate-derived triols in mouse epidermis.***A*, relative abundances of four selected triol isomers in epidermis of wild-type (WT), heterozygous (*Ephx3*^*+/−*^), and homozygous null (*Ephx3*^*−/−*^) *Ephx3*-deficient mice (±SEM, n = 3/genotype, ∗*p* < 0.05 *versus* WT). *B*, overlay of the normal-phase LC-MS analysis of linoleate triols from one representative wild-type (*black*) and representative *Ephx3*^*−/−*^ (*red*) triol profile recorded at m/z 369. The retention times vary slightly because of the long equilibration times of silica HPLC columns with 1% isopropanol in hexane as running solvent. *C*, epoxide hydrolase transformation of the 9*R*,10*R*-epoxy-13*R*-hydroxy product of 12*R*-LOX and eLOX3 involves introduction of oxygen from water with reversal of configuration at C-10 to produce the 9*R*,10*S*,13*R*-triol. R = HO_2_C(CH_2_)_8_ (24).

To examine the potential effects of EPHX3 deficiency on the hydrolysis of linoleate-derived epoxyalcohols, the epidermis from neonatal pups with the three *Ephx3* genotypes was processed in blinded fashion for quantitative analysis of esterified linoleate-derived trihydroxy derivatives. The results of the trihydroxy-linoleate analyses (Fig. 5*A*) showed a striking change in the abundance of the most prominent linoleate triol, 9*R*,10*S*,13*R*-trihydroxy-11*E*-octadecenoate. An overlay of the profile of triols from a wild-type and *Ephx3*^*−/−*^ epidermis helps emphasize the distinct effect of EPHX3 deficiency on this 9*R*,10*S*,13*R*-triol (Fig. 5*B*). This 9*R*,10*S*,13*R*-trihydroxy isomer is formed by epoxide hydrolase-catalyzed hydrolysis from the major epoxyalcohol of the 12*R*-LOX/eLOX3 pathway, 9*R*,10 *R*,13*R*-epoxyalcohol (Fig. 5*C*), and the results clearly implicate EPHX3 in catalyzing this transformation.

The appearance of additional triols in these analyses is partly a reflection of the sodium borohydride step in the analytical procedure, which was included to convert the prominent epoxy-ketones or dihydroxy-ketones of mouse epidermis to epoxy-hydroxy or trihydroxy derivatives, respectively, and produced a mixture of diastereomers in so doing. On normal-phase HPLC of the DMP acetonide PFB esters of linoleate-derived triols, the first two and the last two in the pattern of eight arise from nonenzymatic hydrolysis of a 9*R*,10*R*-epoxy-13*R*-hydroxy isomer and the middle four (chromatographing only as three peaks in this set of analyses) arise from the corresponding 9*R*,10*R*-epoxy-13*S*-hydroxy diastereomer (23, 24, 30). All these extra triols, besides the prominent 9*R*,10*S*,13*R* isomer, are in somewhat similar abundances and are relatively prominent because the sodium borohydride reduction step in the analytical procedure converted any 13-keto analogues to a mixture or 13*R*- and 13*S*-hydroxy isomers: nonenzymatic hydrolysis of these probably accounts for the majority of these other triol isomers detected on LC-MS.

## Discussion

In this study, we identify EPHX3 in the mouse epidermis as responsible for conversion of the 12*R*-LOX/eLOX linoleate-derived epoxyalcohol (9*R*,10*R*-*trans*-epoxy-11*E*-13*R*-hydroxy-octadecenoate) to the corresponding 9*R*,10*S*,13*R*-triol. In the outer barrier layer of the epidermis in which these reactions occur, linoleate is esterified to the ω-hydroxyl of the skin-specific acylceramide EOS (31), and the reactions with 12*R*-LOX, eLOX3, and now, EPHX3, occur on the EOS ester (*cf.* (22, 23, 28)). This activity of EPHX3, acting on a complex ester, is a departure from the previously recognized activities on epoxide free fatty acid substrates (9, 14, 24) and opens up a new vista on the potential involvement of this epoxide hydrolase in pathophysiology.

While the original analysis of the mouse *Ephx3* gene disruption identified no distinctive physiological phenotype, in the current study we found that EPHX3 deficiency is associated with a modest defect on the epidermal permeability barrier as reflected in increased TEWL. Although this is a statistically significant effect, the animals survive with no apparent problems, in contrast to the lethal increases in mouse skin TEWL induced by inactivation of ichthyosis genes (*e.g.*, with 12*R*-LOX, eLOX3, SDR9C7 and many others (19, 20, 22, 32, 33, 34)). This measured effect of *Ephx3* disrupion may reflect a role for the epoxyalcohol-to-triol hydrolysis as a means to fine-tune or limit the flux through the route of epoxyalcohol dehydrogenation to epoxy-ketone, which the SDR9C7 knockout identified as the crucial pathway leading to the formation of an intact permeability barrier (22).

Despite a high capacity for EpOME hydrolysis *in vitro*, EPHX3 does not contribute to EpOME or other EpFA metabolism *in vivo*. While a solubilized EPHX3 may be catalytically capable of EpOME hydrolysis *in vitro*, restricted cellular or membrane-bound localization of EPHX3 may limited access to free EpFA substrates. While cytotoxic, vasoconstrictive, or inflammatory effects of EpOMEs and DiHOMEs might significantly regulate skin pathology, any changes in these linoleate-derived oxylipins are likely caused by changes in P450-mediated EpOME formation or through EPHX1-and/or EPHX2-mediated hydrolysis.

There is a remarkable concordance of expression of 12*R*-LOX, eLOX3, SDR9C7, and EPHX3 in tissues beyond the epidermis. As indicated in the Human Protein Atlas (www.proteinatlas.org), each is expressed in the upper GI tract (tongue, esophagus, forestomach) and located also in bone marrow and lymphoid tissues (and 12*R*-LOX immunoreactivity and catalytic activity were identified previously in human tonsils (35)). In terms of their potential functioning, there are differences in epidermal and oral lipid compositions, including in the keratinized parts of the mouth (palate and gingiva), in which this is an approximately tenfold lower relative abundance of EOS-related ceramide (with tenfold lower content of esterified linoleate) (36). Although there is some barrier to water loss in these regions, it operates at a fraction of the epidermal barrier, such that the oral palate and gingiva have tenfold higher water permeability than epidermis (37), and, significantly, the palatal and gingival epithelium contain very little covalently bound lipid and do not have a corneocyte lipid envelope (38). Currently there is no information on potential oxidative metabolism of the polyunsaturated lipid esters in keratinized oral and upper GI epithelia.

While EPHX3’s enzymatic activities are poorly understood, several studies suggest that EPHX3 plays an important role in tumor suppression. Head and neck cancer subjects with high EPHX3 expression have higher median survival (5.7 years) compared with subjects with low EPHX3 expression (2.1 years) (39). Consistent with this finding, hypermethylation of the *EPHX3* gene expression is associated with reduced EPHX3 expression and poor prognosis in prostate cancer subjects (40). Whether EPHX3 metabolism of linoleic epoxyalcohols confers protection against tumor progression remains to be determined.

This article is the first to identify a biochemical role for EPHX3 *in vivo*. In contrast to its inability to metabolize epoxyfatty acids, our findings establish that EPHX3 hydrolyzes linoleate-derived epoxy-alcohols esterified in skin ceramides. These data suggest that a search is warranted for other esterified epoxides, which may be good substrates for EPHX3 *in vivo*, particularly in tissues where EPHX3 expression is associated with coexpression of 12*R*-LOX pathway enzymes and in scenarios that implicate a role for EPHX3 in tumor suppression.

## Experimental procedures

### Materials

Trihydroxy-octadecenoates and the corresponding [^2^H_4_]9*R*,10*S*,13*R*-trihydroxy standard were prepared as described previously (23, 30). Oxylipins standards were purchased from Cayman Chemical (Ann Arbor, MI).

### Experiments with wild-type and *Ephx*-deficient mice

*Ephx1*^*−/−*^, *Ephx2*^*−/−*^*, and Ephx3*^*−/−*^ mice were previously generated (3, 4, 14). Heterozygous pairs were bred *Ephx3*^*+/−*^ × *Ephx3*^*+/−*^ to obtain wild-type (WT, *Ephx3*^*+/+*^), *Ephx3*^*+/−*^, and *Ephx3*^*−/−*^ littermates. *Ephx*^*+/−*^/*Ephx2*^*+/−*^*/Ephx3*^*−/−*^ × *Ephx*^*+/−*^/*Ephx2*^*+/−*^*/Ephx3*^*−/−*^ breeding was used to obtain *Ephx*^*−/−*^/*Ephx2*^*−/−*^*/Ephx3*^*−/−*^ (*Ephx1/2/3*^*−/−*^) mice. Mice were maintained in 12-h light-dark cycles with *ad libitum* access to water and NIH-31 chow (Envigo, Madison, WI). All animal experiments were performed according to NIH guidelines and were approved by the NIEHS Animal Care and Use Committee.

### Measurement of transepidermal water loss

TEWL was measured using a Tewameter TM300 (Courage & Khazaka, Colonge, Germany). The Tewameter probe was placed against the dorsal skin of postnatal day 2 pups. Each TEWL measurement was the average of five measurements taken over 50 s. Duplicate measurements were averaged to determine TEWL for each pup.

### Quantitative LC-MS analysis of plasma oxylipins

Two-hundred microliter of plasma was obtained during termanal bleed from inferior vena cava of 12-week-old mice anesthetized with pentabarbital. Plasma was mixed with 200 μl 5% methanol/0.1% acetic acid and spiked with internal standards (11,12-EET-d11, 11,12-DHET-d11, PGE_2_-d9, AA-d9, and 15-HETE-d8). Nonesterified plasma oxylipins were isolated by liquid:liquid extraction with 3 ml ethyl acetate, passed through Maestro A phospholipid removal columns (Tecan, Männedorf, Switzerland), washed with 0.9 ml acetonitrile, and dried in a vacuum centrifuge. Oxylipins were reconstituted in 50 μl of 30% ethanol. Oxylipins were assayed on an Utimate 3000 UHPLC equipped with an Xselect CSH C18, 2.1 × 50 mm, 3.5 μm particle column (Waters, Wilford, MA), and a TSQ Quantiva tandem mass spectrometer (Thermo Fisher Scientific, Waltham, MA). Quantification was determined using multiple reaction monitoring and quantified by a blinded observer using TraceFinder (v4.1, ThermoFisher Scientific). Relative response ratios of analytes were compared with standard curves generated with oxylipins purchased from Cayman Chemical (5).

### Quantitative LC-MS analysis of covalently bound CerOS

The epidermal protein pellets from the above analysis were analyzed essentially as previously described (28). Briefly, after additional washes of the protein pellets with MeOH/CHCl_3_ (1:1, v/v) to remove noncovalently bound ceramides, the protein pellets were incubated in 1 M KOH in 95% methanol at room temperature overnight, and the ceramides released from ester linkage were then recovered by Bligh and Dyer extraction. LC-MS analysis used a Waters Alliance 2695 Separations Module (Waters, Milford, MA) and an LTQ linear ion trap mass spectrometer (Thermo-Electron, San Jose, CA) equipped with an *Ion Max* APCI source. The APCI source was operated in positive ion mode, with full-scan spectra (700–900 *m/z*) acquired using the following optimized parameters: N_2_ sheath gas 50 psi; N_2_ auxiliary gas 5 psi; APCI corona current 5 μA; APCI vaporizer temperature 275 °C; capillary temperature 300 °C; capillary offset 35 V; tube lens offset (at 800 *m/z*) 100 V; AGC target ion count 1e^4^; AGC max. inject time 10 ms. Data acquisition and quantitative spectral analysis were done using Thermo-Finnigan Xcalibur v. 2.0.7 SP1 and LTQ Tune v. 2.5.0. The samples for CerOS analysis were dissolved in the normal-phase LC solvent (hexane/isopropanol/glacial acetic acid, 90:10:0.1 v/v/v) and run isocratically on a Luna 5 μ silica column (25 × 0.2 cm) with a solvent of hexane/isopropanol/glacial acetic acid, 90:10:0.1 (v/v/v) at a flow rate of 0.6 ml/min, with retention time for CerOS of approximately 4 min.

### Quantitative LC-MS analysis of oxidized trihydroxy-octadecenoate-containing ceramides in murine epidermis

The method mainly followed the procedure previously described in detail (22). Briefly, back and body skin from wild-type and *Ephx3*-deficient mouse pups (1–2 days old) was treated overnight at 4 °C with Dispase II (Roche Applied Science, 1.5 mg/ml in phosphate buffered saline, pH 7.4), the epidermis was removed, blotted and weighed, then diced and sonicated in MeOH/CHCl_3_ (1:1, v/v), and after centrifugation the solvent was collected and the extraction repeated three further times. The final protein pellet was stored at −80 °C for subsequent analysis of covalently bound ceramides. The pooled MeOH/CHCl_3_ extracts were taken to dryness and loaded and washed through a 0.5 g silica extraction cartridge (Agilent) in CHCl_3_/hexane (1:1), and ceramides and other polar lipids eluted using 16 ml of CHCl_3_/MeOH (2:1, v/v). To convert epoxy-ketones to epoxy-alcohol and any dihydroxy-ketones to triols, the samples from the silica cartridge fractionation are taken to dryness under a stream of N_2_ in a 2 ml Eppendorf tube, redissolved in 50 μl CHCl_3_ followed by addition of 500 μl MeOH, and treated with 50 μl of a freshly prepared methanolic solution of 10 mg/ml NaBH_4_ for 30 min at room temperature. At this point the samples are spiked with d4-triol internal standard (25 ng of [^2^H_4_]9*R*,10*S*,13*R*-trihydroxy-octadecenoic acid in 10 μl MeOH). After evaporation to dryness the lipid esters were hydrolyzed with 0.5 M KOH in a total volume of 500 μl consisting of 100 μl of 10% MeOH in CHCl_3_, 275 μl MeOH, and 125 μl 2M KOH in 20% water in MeOH and left at room temperature overnight in an Eppendorf shaker under argon (23).

The solvents in the KOH-treated samples were adjusted to the proportions of the second phase of a Bligh and Dyer extraction (MeOH:CHCl_3_:H_2_O, 2:2:1.8 by volume, (41)) by addition of 455 μl CHCl_3_ and 394 μl H_2_O, mixed thoroughly and centrifuged, and the lower organic phase removed with the aid of a 1 ml Hamilton syringe and discarded. The top phase was re-extracted using 0.5 ml of “theoretical” Bligh and Dyer lower phase (prepared by mixing solvents in the final Bligh and Dyer proportions) and again discarded. The upper alkaline phase containing the potassium salts of the oxidized fatty acids was reduced in volume to approximately 0.2 ml under N_2_, 1.8 ml water was added, and the still strongly alkaline sample applied to a preconditioned 1 ml (30 mg) Oasis cartridge. After washing with water until the eluate was neutral (this occurs within 1 ml of water wash), the oxidized linoleates and deuterated internal standard were eluted using 1 ml EtOAc. Subsequently the pentafluorobenzyl (PFB) esters were prepared and further derivatized to the dimethoxypropyl (DMP) acetonide derivative for LC-MS analysis of linoleate triols as described (23). The PFB-DMP derivatives were analyzed as the M-PFB ions by APCI-LC-MS using a TSQ Vantage instrument (Thermo Scientific) with the APCI vaporizer temperature set to 500 °C, and the capillary temperature set to 150 °C. The HPLC used a Waters Alliance 2690 system and a Phenomenex Luna 5 μ silica column (25 × 0.2 cm) with a flow rate of 0.6 ml/min and a solvent of hexane/isopropanol in the proportions 100:1 (v/v) for analysis of linoleate triols (PFB, DMP derivative recorded at m/z 369 (d0) and 373 (d4)) (23).

### Statistics

Tracefinder data output was compiled using Excel (Microsoft, Redmond, WA). Graphical output and comparisons between multiple groups were performed by one-way ANOVA and Bonferroni-corrected post-hoc t-tests using Prism version 8.2.1 (GraphPad Software, San Diego, CA).

## Data availability

The data that support the findings of this study are available from the corresponding author upon reasonable request.

## Conflicts of interest

The authors declare that they have no conflicts of interest with the contents of this article.

## References

[1] Fretland A.J., Omiecinski C.J. (2000). Epoxide hydrolases: biochemistry and molecular biology. Chem. Biol. Interact..

[2] Decker M., Arand M., Cronin A. (2009). Mammalian epoxide hydrolases in xenobiotic metabolism and signalling. Arch. Toxicol..

[3] Miyata M., Kudo G., Lee Y.H., Yang T.J., Gelboin H.V., Fernandez-Salguero P., Kimura S., Gonzalez F.J. (1999). Targeted disruption of the microsomal epoxide hydrolase gene. Microsomal epoxide hydrolase is required for the carcinogenic activity of 7,12-dimethylbenz[a]anthracene. J. Biol. Chem..

[4] Sinal C.J., Miyata M., Tohkin M., Nagata K., Bend J.R., Gonzalez F.J. (2000). Targeted disruption of soluble epoxide hydrolase reveals a role in blood pressure regulation. J. Biol. Chem..

[5] Edin M.L., Gholipour Hamedani B., Gruzdev A., Graves J.P., Lih F.B., Arbes S.J., Singh R., Orjuela Leon A.C., Bradbury J.A., DeGraff L.M., Hoopes S.L., Arand M., Zeldin D. (2018). Epoxide hydrolase 1 (EPHX1) hydrolyzes epoxyeicosanoids and impairs cardiac recovery after ischemia. J. Biol. Chem..

[6] Inceoglu B., Schmelzer K.R., Morisseau C., Jinks S.L., Hammock B.D. (2007). Soluble epoxide hydrolase inhibition reveals novel biological functions of epoxyeicosatrienoic acids (EETs). Prostaglandins Other Lipid Mediat..

[7] Wagner K.M., McReynolds C.B., Schmidt W.K., Hammock B.D. (2017). Soluble epoxide hydrolase as a therapeutic target for pain, inflammatory and neurodegenerative diseases. Pharmacol. Ther.

[8] Lord C.C., Thomas G., Brown J.M. (2013). Mammalian alpha beta hydrolase domain (ABHD) proteins: lipid metabolizing enzymes at the interface of cell signaling and energy metabolism. Biochim. Biophys. Acta..

[9] Decker M., Adamska M., Cronin A., Di Giallonardo F., Burgener J., Marowsky A., Falck J.R., Morisseau C., Hammock B.D., Gruzdev A., Zeldin D.C., Arand M. (2012). EH3 (ABHD9): the first member of a new epoxide hydrolase family with high activity for fatty acid epoxides. J. Lipid Res..

[10] Wang W.C., Yang J., Edin M.L., Wang Y.X., Luo Y., Wan D.B., Yang H.X., Song C.Q., Xue W., Sanidad K.Z., Song M.Y., Bisbee H.A., Bradbury J.A., Nan G.J., Zhang J.N. (2019). Targeted metabolomics identifies the cytochrome P450 monooxygenase eicosanoid pathway as a novel therapeutic target of colon tumorigenesis. Cancer Res..

[11] Moghaddam M.F., Grant D.F., Cheek J.M., Greene J.F., Williamson K.C., Hammock B.D. (1997). Bioactivation of leukotoxins to their toxic diols by epoxide hydrolase. Nat. Med..

[12] Kendall A.C., Nicolaou A. (2013). Bioactive lipid mediators in skin inflammation and immunity. Prog. Lipid Res..

[13] Sorokin A.V., Domenichiello A.F., Dey A.K., Yuan Z.X., Goyal A., Rose S.M., Playford M.P., Ramsden C.E., Mehta N.N. (2018). Bioactive lipid mediator profiles in human psoriasis skin and blood. J. Invest. Dermatol..

[14] Hoopes S.L., Gruzdev A., Edin M.L., Graves J.P., Bradbury J.A., Flake G.P., Lih F.B., DeGraff L.M., Zeldin D.C. (2017). Generation and characterization of epoxide hydrolase 3 (EPHX3)-deficient mice. PLoS One.

[15] Ala U., Piro R.M., Grassi E., Damasco C., Silengo L., Oti M., Provero P., Di Cunto F. (2008). Prediction of human disease genes by human-mouse conserved coexpression analysis. PLoS Comput. Biol..

[16] Elias P.M., Menon G.K. (1991). Structural and lipid biochemical correlates of the epidermal permeability barrier. Adv. Lipid Res..

[17] Fischer J. (2009). Autosomal recessive congenital ichthyosis. J. Invest. Dermatol..

[18] Elias P.M., Williams M.L., Holleran W.M., Jiang Y.J., Schmuth M. (2008). Pathogenesis of permeability barrier abnormalities in the ichthyoses: inherited disorders of lipid metabolism. J. Lipid Res..

[19] Takeichi T., Akiyama M. (2016). Inherited ichthyosis: non-syndromic forms. J. Dermatol..

[20] Krieg P., Furstenberger G. (2014). The role of lipoxygenases in epidermis. Biochim. Biophys. Acta.

[21] Muñoz-Garcia A., Thomas C.P., Keeney D.S., Zheng Y., Brash A.R. (2014). The importance of the lipoxygenase-hepoxilin pathway in the mammalian epidermal barrier. Biochim. Biophys. Acta.

[22] Takeichi T., Hirabayashi T., Miyasaka Y., Kawamoto A., Okuno Y., Taguchi S., Tanahashi K., Murase C., Takama H., Tanaka K., Boeglin W.E., Calcutt M.W., Watanabe D., Kono M., Muro Y. (2020). SDR9C7 catalyzes critical dehydrogenation of acylceramides for skin barrier formation. J. Clin. Invest..

[23] Chiba T., Thomas C.P., Boeglin W.E., O'Donnell V.B., Brash A.R. (2016). The precise structures and stereochemistry of trihydroxy-linoleates esterified in human and porcine epidermis and their significance in skin barrier function: implication of an epoxide hydrolase in the transformations of linoleate. J. Biol. Chem..

[24] Yamanashi H., Boeglin W.E., Morisseau C., Davies R.W., Sulikowski G.A., Hammock B.D., Brash A.R. (2018). Catalytic activities of mammalian epoxide hydrolases with *cis* and *trans* fatty acid epoxides relevant to skin barrier function. J. Lipid Res..

[25] Matsuki M., Yamashita F., Ishida-Yamamoto A., Yamada K., Kinoshita C., Fushiki S., Ueda E., Morishima Y., Tabata K., Yasuno H., Hashida M., Iizuka H., Ikawa M., Okabe M., Kondoh G. (1998). Defective stratum corneum and early neonatal death in mice lacking the gene for transglutaminase 1 (keratinocyte transglutaminase). Proc. Natl. Acad. Sci. U. S. A..

[26] Epp N., Furstenberger G., Muller K., de Juanes S., Leitges M., Hausser I., Thieme F., Liebisch G., Schmitz G., Krieg P. (2007). 12R-lipoxygenase deficiency disrupts epidermal barrier function. J. Cell Biol..

[27] Yamamoto H., Hattori M., Chamulitrat W., Ohno Y., Kihara A. (2020). Skin permeability barrier formation by the ichthyosis-causative gene *FATP4* through formation of the barrier lipid omega-O-acylceramide. Proc. Natl. Acad. Sci. U. S. A..

[28] Zheng Y., Yin H., Boeglin W.E., Elias P.M., Crumrine D., Beier D.R., Brash A.R. (2011). Lipoxygenases mediate the effect of essential fatty acid in skin barrier formation: a proposed role in releasing omega-hydroxyceramide for construction of the corneocyte lipid envelope. J. Biol. Chem..

[29] Krieg P., Rosenberger S., de Juanes S., Latzko S., Hou J., Dick A., Kloz U., van der Hoeven F., Hausser I., Esposito I., Rauh M., Schneider H. (2013). Aloxe3 knockout mice reveal a function of epidermal lipoxygenase-3 as hepoxilin synthase and its pivotal role in barrier formation. J. Invest. Dermatol..

[30] Thomas C.P., Boeglin W.E., Garcia-Diaz Y., O'Donnell V.B., Brash A.R. (2013). Steric analysis of epoxyalcohol and trihydroxy derivatives of 9-hydroperoxy-linoleic acid from hematin and enzymatic synthesis. Chem. Phys. Lipids..

[31] Uchida Y., Holleran W.M. (2008). Omega-O-acylceramide, a lipid essential for mammalian survival. J. Dermatol. Sci..

[32] Honda Y., Kitamura T., Naganuma T., Abe T., Ohno Y., Sassa T., Kihara A. (2017). Decreased skin barrier lipid acylceramide and differentiation-dependent gene expression in ichthyosis gene Nipal4 knockout mice. J. Invest. Dermatol..

[33] Hirabayashi T., Anjo T., Kaneko A., Senoo Y., Shibata A., Takama H., Yokoyama K., Nishito Y., Ono T., Taya C., Muramatsu K., Fukami K., Munoz-Garcia A., Brash A.R., Ikeda K. (2017). PNPLA1 has a crucial role in skin barrier function by directing acylceramide biosynthesis. Nat. Commun..

[34] Fischer J., Bourrat E. (2020). Genetics of inherited ichthyoses and related diseases. Acta Derm. Venereol..

[35] Schneider C., Keeney D.S., Boeglin W.E., Brash A.R. (2001). Detection and cellular localization of 12*R*-lipoxygenase in human tonsils. Arch. Biochem. Biophys..

[36] Wertz P.W., Kremer M., Squier C.A. (1992). Comparison of lipids from epidermal and palatal stratum corneum. J. Invest. Dermatol..

[37] Squier C.A., Cox P., Wertz P.W. (1991). Lipid content and water permeability of skin and oral mucosa. J. Invest. Dermatol..

[38] Chang F., Swartzendruber D.C., Wertz P.W., Squier C.A. (1993). Covalently bound lipids in keratinizing epithelia. Biochim. Biophys. Acta.

[39] Bai G.H., Song J.K., Yuan Y.W., Chen Z., Tian Y., Yin X.H., Niu Y.M., Liu J.G. (2019). Systematic analysis of differentially methylated expressed genes and site-specific methylation as potential prognostic markers in head and neck cancer. J. Cell Physiol..

[40] Stott-Miller M., Zhao S.S., Wright J.L., Kolb S., Bibikova M., Klotzle B., Ostrander E.A., Fan J.B., Feng Z.D., Stanford J.L. (2014). Validation study of genes with hypermethylated promoter regions associated with prostate cancer recurrence. Cancer Epidemiol. Biomarks Prev..

[41] Bligh E.G., Dyer W.J. (1959). A rapid method of total lipid extraction and purification. Can. J. Biochem. Physiol..

