# Supplementary material for: Epoxide hydrolase 3 (Ephx3) gene disruption reduces ceramide linoleate epoxide hydrolysis and impairs skin barrier function
Source: J Biol Chem. 2021 Jan 21;296:100198. doi: 10.1074/jbc.RA120.016570 (PMC7948417; doi:10.1074/jbc.RA120.016570)
Supplement: Table S1 [file mmc1.docx]

| Genotype | Actual | Expected | Actual % | Expected % |
| --- | --- | --- | --- | --- |
| *Ephx1^+/+^/Ephx2^+/+^/Ephx3^+/+^* | 18 | 13.42 | 2.1 | 1.56 |
| *Ephx1^+/+^/Ephx2^+/+^/Ephx3^+/-^* | 31 | 26.84 | 3.61 | 3.13 |
| *Ephx1^+/+^/Ephx2^+/+^/Ephx3^-/-^* | 13 | 13.42 | 1.51 | 1.56 |
| *Ephx1^+/+^/Ephx2^+/-^/Ephx3^+/+^* | 26 | 26.84 | 3.03 | 3.13 |
| *Ephx1^+/+^/Ephx2^+/-^/Ephx3^+/-^* | 56 | 53.69 | 6.52 | 6.25 |
| *Ephx1^+/+^/Ephx2^+/-^/Ephx3^-/-^* | 35 | 26.84 | 4.07 | 3.13 |
| *Ephx1^+/+^/Ephx2^-/-^/Ephx3^+/+^* | 18 | 13.42 | 2.1 | 1.56 |
| *Ephx1^+/+^/Ephx2^-/-^/Ephx3^+/-^* | 23 | 26.84 | 2.68 | 3.13 |
| *Ephx1^+/+^/Ephx2^-/-^/Ephx3^-/-^* | 10 | 13.42 | 1.16 | 1.56 |
| *Ephx1^+/-^/Ephx2^+/+^/Ephx3^+/+^* | 27 | 26.84 | 3.14 | 3.13 |
| *Ephx1^+/-^/Ephx2^+/+^/Ephx3^+/-^* | 62 | 53.69 | 7.22 | 6.25 |
| *Ephx1^+/-^/Ephx2^+/+^/Ephx3^-/-^* | 30 | 26.84 | 3.49 | 3.13 |
| *Ephx1^+/-^/Ephx2^+/-^/Ephx3^+/+^* | 53 | 53.69 | 6.17 | 6.25 |
| *Ephx1^+/-^/Ephx2^+/-^/Ephx3^+/-^* | 103 | 107.38 | 11.99 | 12.5 |
| *Ephx1^+/-^/Ephx2^+/-^/Ephx3^-/-^* | 49 | 53.69 | 5.7 | 6.25 |
| *Ephx1^+/-^/Ephx2^-/-^/Ephx3^+/+^* | 27 | 26.84 | 3.14 | 3.13 |
| *Ephx1^+/-^/Ephx2^-/-^/Ephx3^+/-^* | 53 | 53.69 | 6.17 | 6.25 |
| *Ephx1^+/-^/Ephx2^-/-^/Ephx3^-/-^* | 24 | 26.84 | 2.79 | 3.13 |
| *Ephx1^-/-^/Ephx2^+/+^/Ephx3^+/+^* | 10 | 13.42 | 1.16 | 1.56 |
| *Ephx1^-/-^/Ephx2^+/+^/Ephx3^+/-^* | 23 | 26.84 | 2.68 | 3.13 |
| *Ephx1^-/-^/Ephx2^+/+^/Ephx3^-/-^* | 8 | 13.42 | 0.93 | 1.56 |
| *Ephx1^-/-^/Ephx2^+/-^/Ephx3^+/+^* | 23 | 26.84 | 2.68 | 3.13 |
| *Ephx1^-/-^/Ephx2^+/-^/Ephx3^+/-^* | 57 | 53.69 | 6.64 | 6.25 |
| *Ephx1^-/-^/Ephx2^+/-^/Ephx3^-/-^* | 27 | 26.84 | 3.14 | 3.13 |
| *Ephx1^-/-^/Ephx2^-/-^/Ephx3^+/+^* | 12 | 13.42 | 1.4 | 1.56 |
| *Ephx1^-/-^/Ephx2^-/-^/Ephx3^+/-^* | 28 | 26.84 | 3.26 | 3.13 |
| *Ephx1^-/-^/Ephx2^-/-^/Ephx3^-/-^* | 13 | 13.42 | 1.51 | 1.56 |
| Total | 859 | 859 | 100 | 100 |

**Supplemental Table 1. Mendelian distribution of mouse pups from *Ephx1^+/-^/Ephx2^+/-^/Ephx3^+/-^* mating.** 859 consecutive mouse pups from *Ephx1^+/-^/Ephx2^+/-^/Ephx3^+/-^* by *Ephx1^+/-^/Ephx2^+/-^/Ephx3^+/-^* mating were genotyped at weaning. Actual and expected number, and actual and expected percentage of total pups is shown. Chi square analysis revealed normal Mendelian distribution (p=0.958).
